# Functional Analysis of the C-5 Sterol Desaturase PcErg3 in the Sterol Auxotrophic Oomycete Pathogen *Phytophthora capsici*

**DOI:** 10.3389/fmicb.2022.811132

**Published:** 2022-05-10

**Authors:** Weizhen Wang, Tongshan Cui, Fan Zhang, Zhaolin Xue, Borui Zhang, Xili Liu

**Affiliations:** ^1^Department of Plant Pathology, College of Plant Protection, China Agricultural University, Beijing, China; ^2^State Key Laboratory of Crop Stress Biology for Arid Areas, College of Plant Protection, Northwest A&F University, Yangling, China

**Keywords:** *Phytophthora*, sterol, desaturase, development, pathogenicity, external adversity

## Abstract

Although sterols play an important role in most eukaryotes, some oomycetes, including *Phytophthora* spp., have lost the sterol synthesis pathway. Nevertheless, the *ERG3* gene encoding C-5 sterol desaturase in the sterol synthesis pathway is still present in the genomes of *Phytophthora* spp. *Phytophthora capsici*, a destructive pathogen with a broad range of plant hosts, poses a significant threat to the production of agriculture. This study focused on the *ERG3* gene in *P. capsici* (*PcERG3*) and explored its function in this pathogen. It showed that the *PcERG3* gene could be expressed in all tested developmental stages of *P. capsici*, with sporangium and mycelium displaying higher expression levels. A potential substrate of Erg3 (stellasterol) was used to treat the *P. capsici* wild-type strain and a *PcERG3*Δ transformant, and their sterol profiles were determined by GC-MS. The wild-type strain could convert stellasterol into the down-stream product while the transformant could not, indicating that PcErg3 retains the C-5 sterol desaturase activity. By comparing the biological characteristics of different strains, it was found that *PcERG3* is not important for the development of *P. capsici*. The pathogenicity of the *PcERG3*Δ transformants and the wild-type strain was comparable, suggesting that *PcERG3* is not necessary for the interaction between *P. capsici* and its hosts. Further investigations revealed that the *PcERG3*Δ transformants and the wild-type strain displayed a similar level of tolerance to external adversities such as unsuitable temperatures, high osmotic pressures, and intemperate pH, signifying that *PcERG3* is not essential for *P. capsici* to cope with these environmental stresses.

## Introduction

Oomycetes, composed mainly of Peronosporales and Saprolegniales, are a class of eukaryotic microbes that morphologically resemble fungi and often occupy similar niches (Judelson, 2017). However, phylogenetic evidence indicates that they are not true fungi and have a closer relationship with diatoms, brown algae and golden-brown algae (Tyler et al., 2006). Currently, oomycetes include more than 1,200 described species, which can be divided into saprophytes and pathogens based on their lifestyles (Thines, 2018). The distinction between oomycetes and fungi is also evident in their biological, physiological and biochemical traits (Judelson and Blanco, 2005). For example, the sterol synthesis ability of oomycetes significantly differs from fungi. Fungi can produce large amounts of ergosterol, an essential component for membrane integrity and a determining factor of membrane fluidity, permeability, and activity of membrane-associated proteins (Jordá and Puig, 2020). Due to the importance of ergosterol in fungi, many compounds have been developed and applied to combat plant and animal diseases caused by fungi, including sterol synthesis inhibitors (SBI) that target the ergosterol synthesis pathway and polyene drugs (such as amphotericin B) that directly bind to ergosterol (Vale-Silva et al., 2012; Gisi, 2014; Lepesheva et al., 2018). Nevertheless, the capacity of sterol synthesis has evolved independently between Peronosporales and Saprolegniales (Wang et al., 2021). Species in Saprolegniales are sterol autotrophic, and a relatively complete set of genes in the sterol synthesis pathway are present in their genomes (Madoui et al., 2009; Warrilow et al., 2014); in contrast, all known species in Peronosporales are sterol auxotrophic, with necessary genes for sterol synthesis being absent from their genomes. Notably, the SBI fungicides are not effective in controlling the plant diseases caused by Peronosporales pathogens.

*Phytophthora* is a representative oomycete genus that comprises many plant pathogens devastating for agriculture and natural ecosystems (Attard et al., 2008). Previous studies have demonstrated that *Phytophthora* species cannot synthesize sterols and therefore need to recruit exogenous sterols from environment for the normal life cycle (Hendrix, 1970; Gamir et al., 2017). Interestingly, a few genes in the sterol synthesis pathway are still present in their genomes, including a gene encoding the C-5 sterol desaturase (Erg3, EC 1.14.19.20) (Desmond and Gribaldo, 2009; Dahlin et al., 2017; Wang et al., 2021). In canonical sterol synthesis pathways, the Erg3 is responsible for converting delta 7-sterols into delta 5,7-sterols by adding a double bond at the fifth carbon (Desmond and Gribaldo, 2009). In the model plant *Arabidopsis*, the mutation of Erg3 resulted in an accumulation of delta 7-sterols and delayed seed germination (Gachotte et al., 1995). In the animal pathogen *Candida albicans*, Erg3 is critical for pathogenesis during the oral mucosal infection, and mutations that inactivate this protein could lead to azole resistance *in vitro* (Luna-Tapia et al., 2018; Zhou et al., 2018). In the engineering yeast *Saccharomyces cerevisiae*, mutations in Erg3 caused a change in sterol composition from ergosterol to fecosterol, thereby increasing its thermotolerance during ethanol production (Caspeta et al., 2014). However, the specific function of Erg3 in sterol auxotrophic oomycetes has not yet been reported. *Phytophthora capsici*, a destructive heterothallic oomycete pathogen, poses a significant threat to agricultural production with a broad range of plant hosts (Lamour et al., 2012a). In the current study, we characterized the Erg3 protein in *P. capsici* (named PcErg3 hereafter), explored its expression profile, verified its enzyme activity as C-5 sterol desaturase, and investigated its influence on development, pathogenicity, and tolerance to different external adversities of this important pathogen.

## Materials and Methods

### Bioinformatic Analysis of Erg3 Proteins

The *PcERG3* gene together with its flanking sequences was cloned from the *P. capsici* wild-type strain BYA5 with the primers listed in Supplementary Table 1. The coding sequence of *PcERG3* was predicted by the online tool ORF Finder,^1^ which was further confirmed by reverse transcription PCR (RT-PCR). Other Erg3 protein sequences from different organisms were acquired from different databases, including the National Center for Biotechnology Information (NCBI)^2^ database and the Ensembl protists^3^ database. The accession number of each sequence is shown in Supplementary Table 2. The Mega 6.0 software was used to construct the phylogenetic tree (Tamura et al., 2013). The transmembrane domain prediction of the PcErg3 protein was conducted using Protter server^4^ (Vela-Corcía et al., 2019). The 3D structure of PcErg3 was predicted using AlphaFold2 (Cramer, 2021), and analysis of the protein structure was conducted with the PyMOL software (Mooers, 2020).

### Quantitative PCR

Biological materials of *P. capsici* at different developmental stages were collected, including zoospores, cystospores, germ tubes, mycelia (4-day-old) from V8 medium, mycelia (4-day-old) from minimal medium, mycelia with sporangia (4 days in the dark and another 5 days under light), and infection stage (4 days after inoculation on pepper leaves). The minimal medium contains 0.1 g KNO_3_, 0.2 g K_2_HPO_4_, 0.1 g MgSO_4_, 0.1 g CaCl_2_, 0.1 g L-asparagine, 0.05 g L-serine, 4 g glucose, and 1 mL trace elements in 1 L of distilled water. The trace element solution is consisted of 200 mg FeEDTA, 10 mg CuSO_4_, 10 mg MnCl_2_, 10 mg Na_2_MoO_4_, 10 mg Na_2_B_4_O_7_, and 20 mg ZnSO_4_, and 100 mg thiamine hydrochloride in 100 mL distilled water (Jee and Ko, 1997). The SV Total RNA Isolation kit (Promega, Beijing, China) was used to isolate total RNA, and the PrimeScript RT reagent Kit with gDNA Eraser (Takara, Beijing, China) was used to synthesize cDNA. RNA isolation and cDNA synthesis were performed following the protocols recommended by the manufacturers. *Actin* and *WS21* (encoding the 40S ribosomal protein S3A) genes were used as references to normalize the expression level of the target gene (Yan and Liou, 2006). Quantitative PCR (qPCR) was conducted on an ABI7500 sequence detection system (Applied Biosystems, United States) using the SYBR Premix Dimer Eraser kit (Takara, Beijing, China), with primers listed in Supplementary Table 1 and cDNA as templates. The expression level of *PcERG3* relative to the zoospore stage was calculated using the 2^–△△CT^ method (Livak and Schmittgen, 2001). Each stage was evaluated with three technical replicates and the experiment was repeated three times.

### Genome Editing and Cultivation of *Phytophthora capsici*

The *P. capsici* isolate BYA5 was used as a wild-type strain in this study, which was isolated from an infected pepper sample in Gansu Province of China in 2011. A modified CRISPR/Cas9 system for *Phytophthora* was used to delete the *PcERG3* gene, using an oxathiapiprolin-resistance gene (*PcMuORP1*) as a selection marker (Wang et al., 2019). The wild-type strain and transformants were routinely maintained on solid V8 medium at 25°C in the dark. To compare the mycelium growth of transformants and the wild-type strain, mycelial plugs (5 mm in diameter) were cultivated on fresh solid V8 medium with three replicates, and the diameters of colonies were measured in two vertical dimensions before the colonies reached the edge of Petri dishes. A protocol adapted from a previous study (Pang et al., 2013) was used to produce sporangia and zoospores of *P. capsici*. Briefly, *P. capsici* strains were cultivated at 25°C in the dark on solid V8 medium for 4 days, then in a 12 h-light/12 h-dark photoperiod for another 6 days. To evaluate the sporangium production ability of different strains, a microscope with 100 times magnification was used to count all the sporangia in the entire field of vision with three replicates. To release zoospores from sporangia, 10 mL of water was added into the plates, which were then incubated at 4°C for about 30 min and then at room temperature for 30 min. The zoospore suspensions were collected and the concentrations were determined using a hemocytometer with three replicates. To evaluate the cystospore germination ability of different strains, the concentrations of zoospore suspensions were adjusted to around 5 × 10^5^ mL^–1^, which were plated on 1% agar and incubated at 25°C for 3–8 h until most of the wild-type strain had germinated. The cystospore germination rates were calculated by counting the germinated spores out of every 100 cystospores with three replicates. All experiments for biological characteristic evaluation were repeated three times.

### Sterol Composition Analysis

*P. capsici* wild-type strain BYA5 and the representative *PcERG3*Δ transformant KE2-1 were cultured on solid minimal medium without any sterol at least twice before being transferred to the minimal medium modified with 20 μg/mL stellasterol, which was covered with a layer of cellophane (0.02 mm in thickness). Mycelia were collected and dehydrated by freeze-drying, after which the sterols were extracted from 0.1 g dried samples using a method described in a previous study (Wang et al., 2022). The trimethylsilylation of sterols was carried out in 40 μL N,O-bis (trimethylsilyl)-trifluoroacetamide (BSTFA) for each sample, with a water bath at 60 °C for 60 min. For sterol detection, a Gas Chromatography-Mass Spectrometer (GC-MS, Agilent 7890B/7000C) was used, with 1 μL sample being injected onto the column (HP-5 MS UI, 15 m × 0.25 mm × 0.25 μm). The temperature program was as follows: initial temperature 80°C (1 min), an increasing temperature of 12°C/min to 280°C (8 min), and another increasing temperature of 30°C/min to 290°C (5 min). The data were collected in MRM mode, and the following ion pairs (m/z) were used for the detection of different sterols: 470.2 > 343.2 and 343.1 > 253.1 for stellasterol; 470.5 > 380.4 and 470.5 > 365.4 for brassicasterol; and 468.5 > 378.3 and 468.5 > 363.4 for ergosterol.

### Infection Assays

To compare the relative pathogenicity of transformants and the wild-type strain, zoospores and mycelial plugs were inoculated on pepper leaves and *Nicotiana benthamiana* leaves, respectively. For zoospore inoculation, the concentration of different isolates was adjusted to 20,000 zoospores mL^–1^, after which 10 μL of the zoospore suspension liquid was applied to the abaxial side of pepper leaves, with six replicates for each strain. The inoculated leaves were kept in chambers (80% humidity) at 25°C in the dark. After 3 days, the infection lesions were measured. For mycelial plug inoculation, the wild-type strain and transformants were incubated on solid V8 medium at 25°C in the dark, after which the mycelial plugs (5 mm in diameter) with mycelia were cut from the edge of colonies and inoculated on the abaxial side of *N. benthamiana* leaves. The inoculated leaves were kept in chambers (80% humidity) at 25°C in the dark. After 3 days, the disease lesions were measured and the symptoms were observed under ultraviolet light. The infection assays were repeated twice on both pepper and *N. benthamiana* leaves.

### Tolerance of Transformants to Different Adversities

To evaluate the effects of the absence of *PcERG3* on the tolerance of *P. capsici* to environmental stress, the *PcERG3*Δ transformants and the wild-type strain were exposed to environments with different temperatures, high osmotic pressure, intemperate pH, and fungicides. To determine relative temperature sensitivity, the strains were inoculated on solid V8 medium and incubated at different temperatures, including 4, 14, 18, 25, 28, 32, and 37°C, with three replicates for each temperature. The diameters of colonies were measured before they covered all the plates. To quantify sensitivity to osmotic pressure, the strains were inoculated on solid V8 medium modified with either 1 M sorbitol or 0.5 M KCl and were incubated at 25°C in the dark with three replicates. The normal V8 medium was used as a control, and the inhibition ratios were calculated by comparing the diameters of colonies on high osmotic V8 medium and those on the normal V8 medium. To test pH sensitivity, the strains were inoculated on solid V8 medium with intemperate pH (5 and 11) and were incubated at 25°C in the dark, again with three replicates. The normal V8 medium was used as a control, and the inhibition ratios were calculated by comparing the diameters of colonies on acidic or alkaline V8 medium and those on the normal V8 medium. To test the sensitivity of *P. capsici* strains to fungicides, the wild-type strain and a transformant were grown on solid V8 medium supplemented with tebuconazole (5 and 25 μg/mL) or prochloraz (5 and 25 μg/mL). The fungicides were dissolved in dimethyl sulfoxide (DMSO), and V8 medium containing 0.1% DMSO was used as a control. The inhibition ratio was calculated by comparing the colony diameters on medium with and without fungicides. The experiments for adversity tolerance evaluation were repeated twice.

### Statistical Analysis

The DPS software ver. 7.05 was used for statistical analysis. The data collected were used for one-way ANOVA analysis, and differences between mean data were determined using Duncan’s multiple range test at *p* = 0.01.

### Transcriptome Analysis

The wild type strain BYA5 and the transformant KE2-1 were used for transcriptome comparation. These strains were cultured on V8 medium in the dark for 4 days and under light for 4 h to induce sporangium formation before mycelia were collected, with two biological replicates. Total RNA was extracted using Trizol reagent following the manufacturer’s procedure. After quality control and mRNA purification, the mRNA was fragmented into short fragments. The cDNA libraries were constructed by LC-Bio Technology Co., Ltd. (Hangzhou, China), and the 2 × 150 bp paired-end sequencing (PE150) was performed on an Illumina NovaSeq*™* 6000. To get high quality clean reads, reads were further filtered by Cutadapt.^5^ The filtered reads were aligned to the genome of *P. capsici*^6^ using HISAT2 package (Lamour et al., 2012b; Kim et al., 2019). The mapped reads of each sample were assembled using StringTi (Pertea et al., 2015) before a comprehensive transcriptome was constructed using GffCompare software.^7^ The FPKM (fragment per kilobase of transcript per million mapped reads) value was calculated to estimate the expression levels. Differential expression analysis was performed by DESeq2 software between two different groups (Love et al., 2014). The genes with the parameter of false discovery rate (FDR) below 0.05 and absolute fold change ≥ 2 were considered differentially expressed genes (DEGs). The Pearson correlation coefficient between two samples was calculated to evaluate repeatability between replicas, and Gene ontology (GO) enrichment analysis was conducted for functional classification of DEGs.

## Results

### Characterization of Erg3 and Its Expression Profile in *Phytophthora capsici*

**FIGURE 1:**
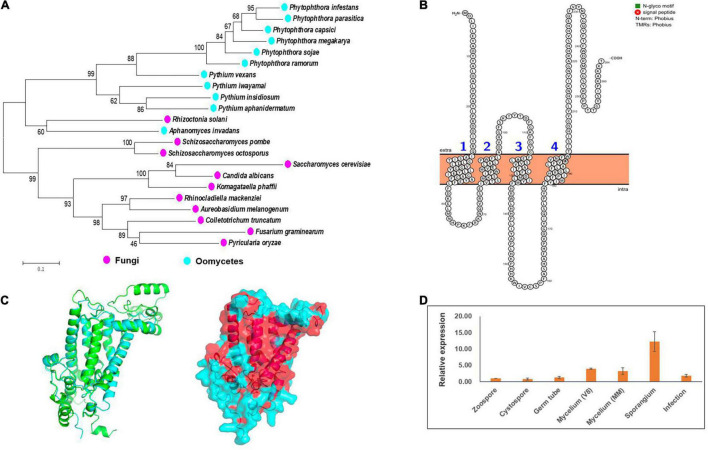
Characterization of Erg3 protein and its expression profile in *P. capsici*. **(A)** Molecular phylogenetic tree of Erg3 protein sequences of species from different oomycetes and fungi. Bootstrap values are expressed as percentages based on 1,000 repetitions. **(B)** Four transmembrane domains are predicted to be present in the PcErg3 protein with Protter. **(C)** The 3D structure analysis of PcErg3. Left: The alignment of 3D structures of Erg3 from *P. capsici* (blue) and that from *S. cerevisiae* (green), which were both predicted with AlphaFold2; right: the prediction of hydrophobic regions (red) of PcErg3 with AlphaFold2. **(D)** Expression profile of *PcERG3* at different stages in *P. capsici*. The gene *PcERG3* is expressed in all development stages and during infection (4 dpi). V8 means that mycelia were cultured on V8 medium, and MM indicates that mycelia were cultured on minimal medium without any sterol. Values represent mean ± *SD* from three independent biological repeats.

By exploiting genome data analysis, it was found that the *ERG3* gene is present in *Phytophthora* and *Pythium* spp. (Desmond and Gribaldo, 2009; Dahlin et al., 2017; Wang et al., 2021). We tried to clone this gene based on the genome information of the strain LT1534 (see text footnote 6) in JGI database, but it was not successful. The first half part of this gene together with it upstream could be amplified, but not the second half part. Therefore, we speculated that it had a wrong gene model in JGI database. We then designed forward primer based on the genome information, and designed reverse primer based on the relatively conserved region of other *Phytophthora* species (*P. sojae* and *P. ramorum*), amplifying the *PcERG3* together with its flanking sequences with DNA of the wild-type *P. capsici* strain BYA5 as template. With Sanger sequencing, the long sequence containing *PcERG3* was obtained. ORF Finder was used to predict the coding sequence of this gene (GenBank accession number: OM571178), which identified a protein with a high similarity to Erg3 in other *Phytophthora* species (Figure 1A). Phylogenetic analysis of Erg3 proteins from different organisms also showed that they are relatively conserved within oomycetes and oomycete Erg3 are closely related to fungal Erg3 (Figure 1A). This could be further validated by the high similarity between the predicted 3D structures of PcErg3 and Erg3 from *S. cerevisiae* (Figure 1C). The *PcERG3* gene was found to consist of 795 nucleotides and encode a protein that is 264 amino acids in length. The predicted *PcERG3* gene was cloned from the wild-type *P. capsici* isolate BYA5 using the primers listed in Supplementary Table 1, with both DNA and cDNA as templates (Supplementary Figure 1). Sanger sequencing was performed to further confirm the gene model. Protein signature analysis of PcErg3 from *P. capsici* identified four transmembrane domains (Figure 1B) and 3D structure analysis identified large hydrophobic regions (Figure 1C), indicating that it is likely a transmembrane protein.

Since *P. capsici* is sterol auxotrophic and lacks the *de novo* sterol synthesis pathway, it is important to determine whether *PcERG3* is expressed in *P. capsici*. The qPCR was conducted using RNA isolated from different life stages. To determine whether the expression of *PcERG3* is regulated by exogenous sterols, its expression levels were compared between mycelia grown on V8 medium that is made from vegetable juice containing natural plant sterols and mycelia grown on minimal medium without sterol. The results showed that *PcERG3* was expressed in all tested developmental stages and during infection, while the expression level in zoospore and cystospore was much lower compared to other stages. The sporangial stage showed the highest expression level, and there was no difference in expression level of *PcERG3* between mycelia grown on the medium with- or without plant sterols (Figure 1D).

### PcErg3 Retains C-5 Sterol Desaturase Activity in *Phytophthora capsici*

**FIGURE 2:**
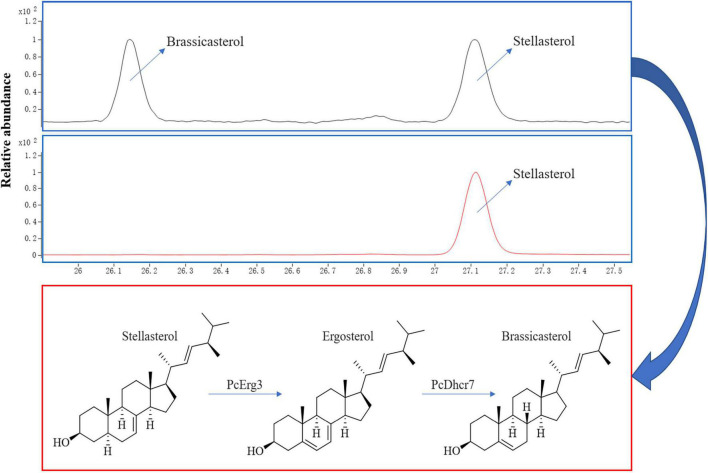
Chromatograms for sterol detection from different *P. capsici* strains and the pathway for sterol conversion in the wild-type strain. Blue boxes represent sterol detection chromatograms for the wild-type strain BYA5 (above) and the *PcERG3*Δ transformant KE2-1 (bottom), which were both cultured on minimal medium modified with 20 μg/mL stellasterol. All the sterols indicated in the figure were detected as sterol derivatives with a trimethylsilyl at the C-3 hydroxyl. Red box represents the sterol conversion of stellasterol in the wild-type strain of *P. capsici* mediated by the enzymes in sterol biosynthesis pathway it harbors.

Using a modified CRISPR/Cas9 system for *Phytophthora* species, we successfully deleted the *PcERG3* gene in *P. capsici* and obtained three homozygous transformants (Wang et al., 2019). To determine whether the protein encoded by *PcERG3* has the C-5 sterol desaturase activity, stellasterol, a delta-7 sterol and potential substrate of Erg3, was used to treat the wild-type strain (BYA5) and a representative transformant (KE2-1). The mycelia of both strains were collected, and the sterols were extracted and characterized. As a result, stellasterol and brassicasterol were detected in the wild-type strain, while only stellasterol was present in the transformant (Figure 2). In theory, the downstream product of stellasterol by Erg3 is ergosterol; however, ergosterol was found in neither the wild-type strain nor the transformant. This result reflects findings from our previous study, which demonstrated that *P. capsici* also has a Dhcr7 protein (PcDhcr7) that efficiently converts ergosterol into brassicasterol (Wang et al., 2022). Taken together, the PcErg3 protein retains the C-5 sterol desaturase activity in *P. capsici* without homologous proteins, and it enables the conversion of stellasterol into ergosterol, which is subsequently converted into brassicasterol by PcDhcr7 (Figure 2).

### Influence of Loss of *PcERG3* on Development of *Phytophthora capsici*

Even though PcErg3 has the C-5 sterol desaturase activity, the importance of this protein for the life cycle of *P. capsici* remains unknown. To address this question, we compared the biological characteristics of the transformants to those of the wild-type strain, including mycelium growth, sporangium production, zoospore production, and cystospore germination. The results showed that the mycelium growth of *P. capsici* was not affected after the *PcERG3* gene was deleted. The sporangium production increased in the *PcERG3*Δ transformants, while the zoospore production was comparable in transformants and the wild-type strain. Most of the cystospores of transformants could germinate normally, and there was no significant difference in the rate of cystospore germination between transformants and the wild-type strain (Table 1). Interestingly, the transformants could produce more sporangia even though the expression level of *PcERG3* is relatively high at this developmental stage. It is possible that PcErg3 has a negative role in sporangium differentiation and production; however, the total zoospore production was not promoted in the transformants, implying a possible positive role of PcErg3 in zoospore differentiation or zoospore release. Overall, the loss of the *PcERG3* gene did not distinctly impair the development of *P. capsici*.

**TABLE 1:** Biological characteristics of wild-type strain and *PcERG3*Δ transformants.

Strain	Colony diameter (mm)	Sporangium production	Zoospore release (× 10^4^/mL)	Cystospore germination rate (%)
BYA5	67.00 ± 0.87	174.00 ± 14.20	15.33 ± 1.21	86.56 ± 3.17
KE1-1	67.14 ± 2.35	265.89 ± 9.26**	16.00 ± 2.02	88.55 ± 5.21
KE2-1	66.05 ± 0.63	240.45 ± 3.67**	15.50 ± 1.83	87.44 ± 2.34
KE3-1	66.67 ± 0.17	251.67 ± 2.85**	14.06 ± 1.13	–

Values represent mean ± SD of three independent biological repeats; “–” indicates the data is not determined; and double asterisks denote significant difference from wild-type strain BYA5. **p < 0.01.

### Pathogenicity of *PcERG3*Δ Transformants of *Phytophthora capsici*

**FIGURE 3:**
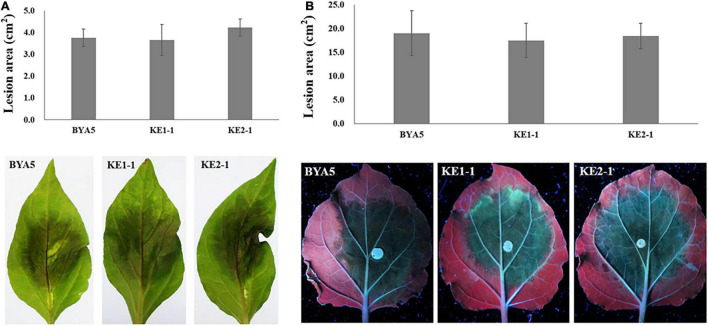
Pathogenicity of wild-type strain and *PcERG3*Δ transformants on pepper and *N. benthamiana* leaves. **(A)** Lesion area (above) and symptoms (bottom) on pepper leaves inoculated with zoospores of different strains (3 dpi). **(B)** Lesion area (above) and symptoms (bottom) on *N. benthamiana* leaves inoculated with mycelial plugs of different strains (3 dpi). BYA5 is the wild-type strain; KE1-1 and KE2-1 are representative *PcERG3*Δ transformants. Values represent mean ± *SD* from six replicates from one experiment. The experiments were repeated twice with similar results.

The interaction between *Phytophthora* spp. and plants is a complex process involving in a variety of players and pathways (Naveed et al., 2020). To explore whether PcEer3 mediates a role in this interaction, the pathogenicity was compared between the *PcERG3*Δ transformants and the wild-type strain. The zoospores of transformants (KE1-1 and KE2-1) and the wild-type strain (BYA5) were inoculated on the leaves of pepper, and the mycelial plugs of transformants and the wild-type strain were inoculated on the leaves of *N. benthamiana*. The results of these experiments showed that the pathogenicity of transformants was comparable to that of the wild-type strain in both cases (Figures 3A,B), indicating that the *PcERG3* gene is not important for the pathogenicity of *P. capsici.*

### Tolerance of *PcERG3*Δ Transformants of *Phytophthora capsici* to Environmental Stress

**FIGURE 4:**
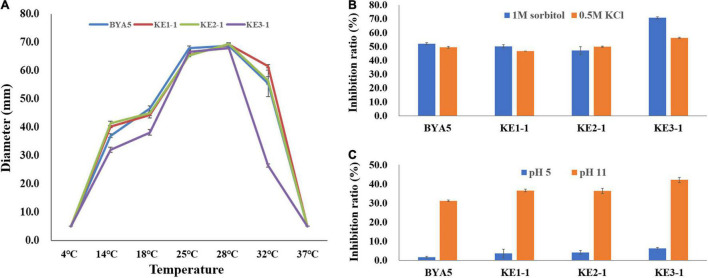
Tolerance of wild-type strain and *PcERG3*Δ transformants to different external adversities. **(A)** Colony growth of different strains under a series of temperatures. The diameters were measured after 4 days of incubation in the dark. **(B)** Inhibition ratios of high osmotic pressures (1 M sorbitol and 0.5 M KCl) on colony growth of different strains. **(C)** Inhibition ratios of intemperate pH (pH 5 and pH 11) on colony growth of different strains. BYA5 is the wild-type strain; KE1-1, KE2-1, and KE3-1 are the *PcERG3*Δ transformants. Values represent mean ± *SD* from three replicates from one experiment. The experiments were repeated twice with similar results.

Plant pathogens may be confronted with a variety of external stressors throughout their life cycles, and tolerance to these stressors largely determines the field fitness of pathogens (Chung, 2012). To verify whether PcErg3 has a role in the field fitness of *P. capsici*, we evaluated the relative sensitivities of the *PcERG3*Δ transformants and the wild-type strain to alterations in environmental factors, including temperature, osmotic pressure, and pH. The temperature sensitivity test showed that transformants and the wild-type strain displayed similar growth patterns under a series of temperatures, with the exception of KE3-1. All the transformants and the wild-type strain grew fast at ambient temperatures between 25 and 28°C. Under higher or lower temperatures, all strains grew slower, and the growth was halted completely for all strains under 4 and 37°C (Figure 4A). To simulate high osmotic pressure conditions, plates containing V8 medium with either 1 M sorbitol or 0.5 M KCl were used for the cultivation of the *PcERG3*Δ transformants and the wild-type strain. These high osmotic pressure conditions inhibited the growth of transformants and the wild-type strain at a similar level with inhibition ratios around 50%, again with the exception of KE3-1 (Figure 4B). To evaluate how pH influences transformants and the wild-type strain, the pH value of the V8 medium was adjusted to 5 or 11. The pH 5 condition barely inhibited all strains with inhibition ratios less than 7%, while the pH 11 condition inhibited the growth of two transformants (KE1-1 and KE2-1) and the wild-type strain by 31–37% (Figure 4C). It seems that the transformant KE3-1 is more sensitive to the pH 11 condition, with an inhibition ratio of 42.21% (Figure 4C). From the above results, it can be concluded that the PcErg3 protein is not essential for *P. capsici* to cope with environmental stressors such as low or high temperature, high osmotic pressure, and intemperate pH. In fungi, Erg3 is known to play a key role in sterol synthesis. Therefore, the tolerance of the wild-type strain and a *PcERG3*Δ transformant were used for the sensitivity test to sterol biosynthesis inhibitors (tebuconazole and prochloraz). The results showed that these fungicides could hardly inhibit the growth of the transformant and the wild-type strain even at high concentrations (Supplementary Figure 2). This is as expected, because *Phytophthora* is sterol auxotrophic and lacks the sterol synthesis pathway.

### Transcriptome Comparation of *PcERG3*Δ Transformant and Wild-Type Strain

**FIGURE 5:**
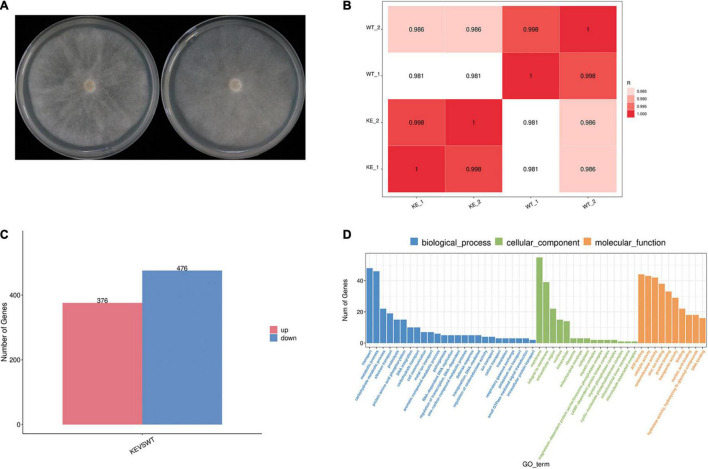
Transcriptome comparation between a *PcERG3*Δ transformant and the wild-type strain of *P. capsici*. **(A)** Colonies of the wild-type strain BYA5 (left) and the *PcERG3*Δ transformant KE2-1 (right) used for RNA-Seq. **(B)** Pearson correlation between samples. WT_1 and WT_2 are two biological replicates of BYA5; KE_1 and KE_2 are two biological replicates of KE2-1. **(C)** The number of up- and down-regulated genes in transcriptome of KE2-1 (KE) in comparison to that of BYA5 (WT). **(D)** Gene ontology (GO) annotation of genes that showed changes in expression in KE2-1 in comparison to BYA5. The number of genes with different GO terms is shown in the three GO categories: biological process, cellular component and molecular function.

To further address the question whether the loss of *PcERG3* has an influence on *P. capsici*, RNA-Seq was performed with mycelia during sporangium production (Figure 5A). The transcriptome comparation was conducted between the *PcERG3*Δ transformant KE2-1 and the wild-type strain BYA5. The results showed that the transcriptomes of two different biological replicates from the same strain were similar between each other; however, the transcriptome of KE2-1 was distinctly different from that of BYA5 (Figure 5B). Among approximate 14,400 genes that were found to be expressed at this stage, 852 genes were significantly changed in their expression level, with 376 genes being up-regulated and 476 genes being down-regulated in the knock-out transformant in comparison to the wild-type strain (Figure 5C). Gene ontology (GO) analysis indicated that many differentially expressed genes (DEGs) participate in several important biological processes, including transport, metabolic process, carbohydrate metabolic process, and electron transport. From another perspective, DEGs encode proteins belonging to different components, with membrane, integral to membrane, and extracellular region being the most predominant (Figure 5D). Proper transportations of membrane related proteins are important for zoospore differentiation, and this may partially explain why zoospore production was not significantly increased while much more sporangia were formed in the *PcERG3*Δ transformants.

## Discussion

Sterols are an important class of lipids with essential roles in the cells of most eukaryotes. However, some eukaryotic lineages, including some oomycetes, have lost the ability of *de novo* sterol synthesis (Desmond and Gribaldo, 2009). Although it remains unclear what factors drove the loss of genes in the sterol synthesis pathway, the composition of host sterols may be an important mechanism, as demonstrated by the increased dependence of sterol auxotrophic oomycetes on their hosts compared to sterol autotrophic oomycetes (Wang et al., 2021). In spite of an incomplete pathway in certain organisms, some of the underlying genes may have been retained, often with significant evolutionary changes. For example, *Caenorhabditis elegans* lacks some of the important players of Hedgehog signaling but retains the *PATCHED* genes, one of which could play a key role in germ-line cytokinesis (Kuwabara et al., 2000). Based on bioinformatic analyses, it was found that Erg3 proteins from oomycetes are phylogenetically close to those from fungi; additionally, PcErg3 is a transmembrane protein that has a high similarity of 3D structure with its homolog from *S. cerevisiae*, indicating that it may also be located in the endoplasmic reticulum like in the yeast *S. cerevisiae* (Nishino et al., 1981). A previous study using the *PcERG3* gene to complement the yeast *ERG3* null mutant found that the expression of *PcERG3* gene in the yeast mutant could largely rescue its ergosterol synthesis ability, implying the C-5 sterol desaturase activity of PcErg3 in *S. cerevisiae* (Nesnow, 2013). However, it is not known whether PcErg3 is actually expressed in *P. capsici*, and if so, what function it serves in this pathogen.

This study used a potential precursor substance of Erg3 to treat a *PcERG3*Δ transformant and the wild-type strain of *P. capsici* and determined their sterol compositions. The results showed that the wild-type strain could convert the substance into the product, but not the *PcERG3*Δ transformant. This indicates that *PcERG3* is expressed and translated into a protein that retains the C-5 sterol desaturase activity. Investigations into biological characteristics and pathogenicity showed that the *PcERG3* gene is not essential for the development of *P. capsici* or its interaction with plant hosts. All the existing organisms have been shaped by their evolutionary history, and the evolution is always ongoing. It is possible that some organisms have a few redundant remnants of evolution, but in theory, any full redundancy should be evolutionarily unstable (Thomas, 1993). Given that the *ERG3* gene is relatively conserved in different auxotrophic oomycetes, including species of *Phytophthora* and *Pythium*, it is less likely that *PcERG3* is a fully functional redundant gene in *P. capsici*. We speculated that PcErg3 plays a role in some special circumstances, and the relative tolerances of the *PcERG3*Δ transformants and the wild-type strain to different environmental stressors were compared. Even though the *PcERG3* gene is not important for *P. capsici* to cope with unfavorable temperatures, high osmotic pressure, or intemperate pH, the role of this gene cannot be ruled out in other particular cases, particularly because field environments are much more complex than the laboratory conditions (Fang et al., 2018). Another possibility is that Erg3 together with Dhcr7 in *Phytophthora* spp. could transform inactive sterols recruited from plants into active ones to benefit self-development in some special situations, given that *Phytophthora* spp. have a preference for Δ5 sterols (Knights and Elliott, 1976; Wang et al., 2022), and that the sterol composition in plants can be variable among different species and may change upon external stimulus (Griebel and Zeier, 2010; Valitova et al., 2016).

Even though no apparent difference was found between the *PcERG3*Δ transformant KE2-1 and the wild-type strain, their transcriptomes were distinctly different, with many DEGs being identified. This suggests that the transcriptome of *Phytophthora* can be very plastic, as reported in other organisms (Vesga et al., 2020; Dang et al., 2021). This plasticity may enable *Phytophthora* spp. to adapt to different environments or circumstances such as the loss of function of certain genes (Wang et al., 2022). Therefore, the transcriptome remodeling is likely also playing a key role in compensating the lack of *PcERG3* in *P. capsici*. Among the three homozygous transformants, KE3-1 seemed to be distinct from the other two in phenotype. This observation may have been resulted from an off-target effect of the CRISPR/Cas9 or side effects of the transformation process (Zhang et al., 2015; Friedman et al., 2018). To further reveal the biological function of PcErg3 in depth, more investigations are needed, and this could be facilitated by the utilization of various methods including omics analysis, cell biological research, and biochemical comparation.

## Conclusion

In conclusion, the current study found that *P. capsici* harbors a single copy of the *PcERG3* gene, which could be expressed in all tested developmental stages of this pathogen, with sporangium and mycelium displaying higher expression levels. By treating the wild-type strain and a representative *PcERG3*Δ transformant of *P. capsici* with a potential substrate sterol (stellasterol), it was found that the wild-type strain could transform the substrate to the down-stream product, but not the *PcERG3*Δ transformant, thus demonstrating the molecular function of PcErg3 protein as a C-5 sterol desaturase. By comparing the phenotypes of the *PcERG3*Δ transformants with those of the wild-type strain, it showed that *PcERG3* in *P. capsici* is not essential for development, pathogenicity, or tolerance to environmental stressors.

## Data Availability Statement

The original contributions presented in the study are included in the article/Supplementary Material, further inquiries can be directed to the corresponding author/s.

## Author Contributions

XL and WW conceived and designed the experiments. WW, TC, FZ, ZX, and BZ performed the experiments. WW, TC, FZ, ZX, BZ, and XL contributed to reagents, materials, and analysis tools. XL supervised the work. WW wrote the main manuscript. XL and TC revised the manuscript. All authors have read and approved the final manuscript.

## Conflict of Interest

The authors declare that the research was conducted in the absence of any commercial or financial relationships that could be construed as a potential conflict of interest.

## Publisher’s Note

All claims expressed in this article are solely those of the authors and do not necessarily represent those of their affiliated organizations, or those of the publisher, the editors and the reviewers. Any product that may be evaluated in this article, or claim that may be made by its manufacturer, is not guaranteed or endorsed by the publisher.

## Footnotes

1
http://www.bioinformatics.org/sms2/orf_find.html

2
https://www.ncbi.nlm.nih.gov

3
https://protists.ensembl.org/index.html

4
http://wlab.ethz.ch/protter/start/

5
https://cutadapt.readthedocs.io/en/stable/

6
https://phycocosm.jgi.doe.gov/Phyca11/Phyca11.home.html

7
http://ccb.jhu.edu/software/stringtie/gffcompare.shtml
