# Supplementary material for: Functional Analysis of the C-5 Sterol Desaturase PcErg3 in the Sterol Auxotrophic Oomycete Pathogen Phytophthora capsici
Source: Front Microbiol. 2022 May 10;13:811132. doi: 10.3389/fmicb.2022.811132 (PMC9151008; doi:10.3389/fmicb.2022.811132)
Supplement: Supplementary file 1 [file Data_Sheet_1.docx]

**Supplementary information**

**Table S1.** Primers used in this study.

| **Primer name** | **Primer sequence (5′-3′)** | **Purpose** |
| --- | --- | --- |
| E-ALL-F | GCCAAATGACGGAACAAAG | Amplifying and sequencing *PcERG3* together with its flanking sequences |
| E-ALL-R | TGGGAGAGAACGGGACTAT | Amplifying and sequencing *PcERG3* together with its flanking sequences |
| ERG3-F | ATGGACCTCATCCTGGAATAC | Amplifying and sequencing *PcERG3* |
| ERG3-R | CTATGTTTTCTTTGCCTTGGGT | Amplifying and sequencing *PcERG3* |
| qERG-F | GCCAGATTGCATCCATCAC | qPCR for *PcERG3* gene |
| qERG-R | CTCAAACCCGTAGCCGAAC | qPCR for *PcERG3* gene |
| Actin-F | ACTGCACGTTCCAGACGATC | qPCR for *Actin* gene |
| Actin-R | CCACCACCTTGATCTTCATG | qPCR for *Actin* gene |
| WS21-F | GGAAAGAACAAACGCCTGAC | qPCR for *WS21* gene |
| WS21-R | GTTGCGCTCCGAGAAGATA | qPCR for *WS21* gene |

**Table S2.** Accession numbers of the Erg3 sequences of different organisms.

| **Species** | **Database** | **Accession number** |
| --- | --- | --- |
| *Rhinocladiella mackenziei* | NCBI | XP_013277621.1 |
| *Saccharomyces cerevisiae* | NCBI | NP_013157.1 |
| *Phytophthora megakarya* | NCBI | OWZ01640.1 |
| *Phytophthora parasitica* | Ensembl protists | ETP13889 |
| *Phytophthora ramorum* | Ensembl protists | Phyra72427 |
| *Pythium aphanidermatum* | Ensembl protists | EPrPAT00000015118 |
| *Pythium vexans* | Ensembl protists | EPrPVT00000023047 |
| *Pythium iwayamai* | Ensembl protists | EPrPWT00000023819 |
| *Aureobasidium melanogenum* | NCBI | KAG9762428.1 |
| *Fusarium graminearum* | NCBI | XP_011318434.1 |
| *Pyricularia oryzae* | NCBI | ELQ69078.1 |
| *Colletotrichum truncatum* | NCBI | XP_036584870.1 |
| *Candida albicans* | NCBI | AOW26151.1 |
| *Schizosaccharomyces pombe* | NCBI | NP_593135.1 |
| *Schizosaccharomyces octosporus* | NCBI | XP_013016056.1 |
| *Saccharomyces cerevisiae* | NCBI | NP_013157.1 |
| *Komagataella phaffii* | NCBI | XP_002490491.1 |
| *Rhizoctonia solani* | NCBI | EUC64856.1 |
| *Pythium insidiosum* | NCBI | GAY03298.1 |
| *Phytophthora sojae* | Ensembl protists | EGZ27765 |
| *Phytophthora infestans* | Ensembl protists | PITG_21426T0 |
| *Aphanomyces invadans* | Ensembl protists | RHY25242 |


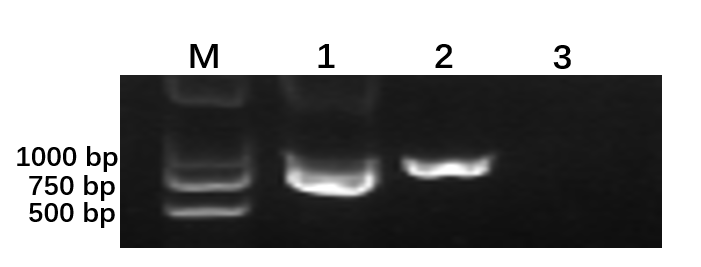


**Figure S1.** Gene model confirmation with PCR and gel electrophoresis. The gene *PcERG3* was amplified from *P. capsici* strain BYA5 with both DNA and cDNA as templates. M, marker; lane 1 indicates the PCR product with DNA as template; lane 2 indicates the PCR product with cDNA as template; lane 3 indicates blank control. Sanger sequencing showed that the products from lane 1 and lane 2 were same.


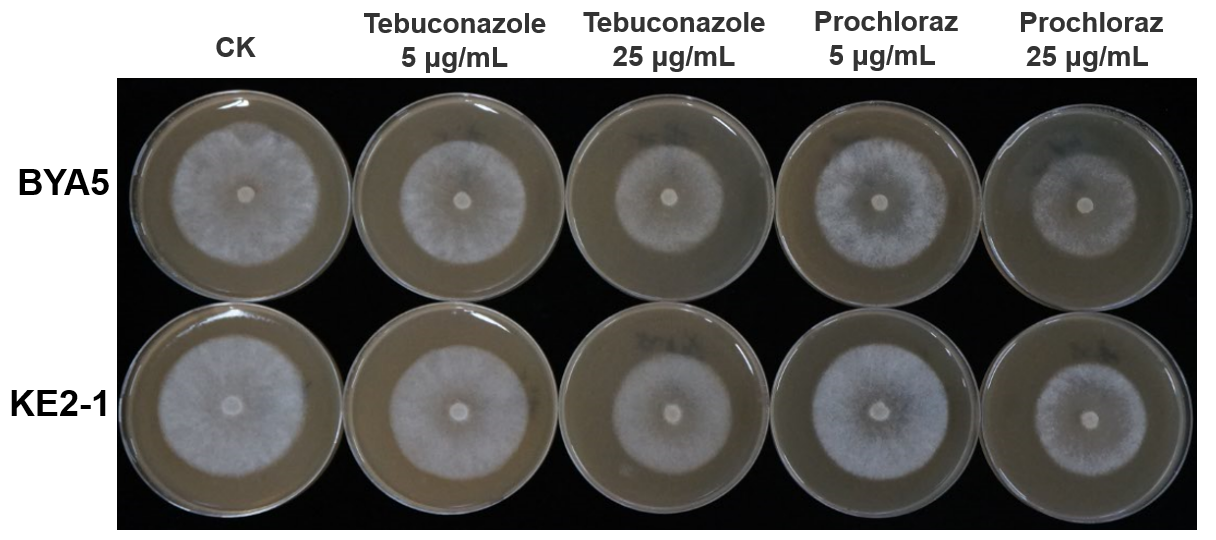


**Figure S2.** Tolerance of a *PcERG3*Δ transformant and the wild-type strain of *P. capsici* to sterol biosynthesis inhibitors. BYA5 is the wild-type strain; KE2-1is a *PcERG3*Δ transformant. V8 medium modified with different concentrations of demethylation inhibitors (as indicated on the top of the figure) was used for the culturation of *P. capsici* strains*.* CK indicates the control without fungicides. The experiment was repeated twice with similar results.
